# Supplementary material for: Kidney Measurement and Glomerular Filtration Rate Evolution in Children with Polycystic Kidney Disease
Source: Children (Basel). 2024 May 10;11(5):575. doi: 10.3390/children11050575 (PMC11119250; doi:10.3390/children11050575)
Supplement: Supplementary file 1 [file children-11-00575-s001.zip › children-3005009-supplementary.pdf]

Supplemental table S1. Glomerular filtration rates in all children from the initial evaluation until the 1-year follow-up.

Age	Brion eGFR	Schwartz eGFR	Quadratic eGFR	Follow-up Schwartz eGFR	Follow-up Quadratic eGFR	PKD mutation
0.08	46.20314279	-	-	152.8060841	121.6342567	PKD1
0.5	69.09018036	-	-	136.0218171	117.5222531	PKD1
2	-	143.782977	120.4903275	178.2517226	123.5248596	-
3	-	162.8206727	124.1637745	N/A	N/A	PKD2
5	-	136.3488321	115.4855086	149.5052984	119.5878744	-
7	-	160.2625648	125.8456194	160.9295409	121.760026	PKD1
8	-	135.8253194	116.7218252	113.8954732	106.8699015	PKD1
9	-	133.8804325	120.6295089	153.6194308	125.9122352	PKD1
9	-	105.1544704	106.0002908	N/A	N/A	PKD1
12	-	195.5394414	125.536702	N/A	N/A	PKD2
12	-	115.2070761	109.3185317	114.1974314	108.8859442	PKD2
15	-	100.4387047	105.6900692	N/A	N/A	-
15	-	76.92247672	86.49234885	N/A	N/A	PKD2
16	-	79.12699184	89.00341578	N/A	N/A	PKD2
16	-	115.3778217	115.4869372	N/A	N/A	PKD2
16	-	107.2655515	106.5189492	N/A	N/A	-

Legend: Age in years; eGFR=estimated glomerular filtration measured in ml/min/1.73m^2; N/A=not assigned.
